# Differentiating Progressive Supranuclear Palsy and Parkinson's Disease With Head-Mounted Displays

**DOI:** 10.3389/fneur.2021.791366

**Published:** 2021-12-23

**Authors:** Arvid Herwig, Almedin Agic, Hans-Jürgen Huppertz, Randolf Klingebiel, Frédéric Zuhorn, Werner X. Schneider, Wolf-Rüdiger Schäbitz, Andreas Rogalewski

**Affiliations:** ^1^Department of Psychology, Clinical Psychology and Psychotherapy, University of Bremen, Bremen, Germany; ^2^Department of Psychology, Neuro-Cognitive Psychology, and Cognitive Interaction Technology (CITEC), Bielefeld University, Bielefeld, Germany; ^3^Department of Neurology, Evangelisches Klinikum Bethel, University Hospital OWL of the University Bielefeld, Bielefeld, Germany; ^4^Swiss Epilepsy Clinic, Klinik Lengg, Zürich, Switzerland; ^5^Department of Neuroradiology, Evangelisches Klinikum Bethel, University Hospital OWL of the University Bielefeld, Bielefeld, Germany

**Keywords:** oculomotor, head-mounted display, neurodegenerative disease, progressive supranuclear palsy, Parkinson's disease, saccades

## Abstract

**Background:** Progressive supranuclear palsy (PSP) is a neurodegenerative disorder that, especially in the early stages of the disease, is clinically difficult to distinguish from Parkinson's disease (PD).

**Objective:** This study aimed at assessing the use of eye-tracking in head-mounted displays (HMDs) for differentiating PSP and PD.

**Methods:** Saccadic eye movements of 13 patients with PSP, 15 patients with PD, and a group of 16 healthy controls (HCs) were measured. To improve applicability in an inpatient setting and standardize the diagnosis, all the tests were conducted in a HMD. In addition, patients underwent atlas-based volumetric analysis of various brain regions based on high-resolution MRI.

**Results:** Patients with PSP displayed unique abnormalities in vertical saccade velocity and saccade gain, while horizontal saccades were less affected. A novel diagnostic index was derived, multiplying the ratios of vertical to horizontal gain and velocity, allowing segregation of PSP from PD with high sensitivity (10/13, 77%) and specificity (14/15, 93%). As expected, patients with PSP as compared with patients with PD showed regional atrophy in midbrain volume, the midbrain plane, and the midbrain tegmentum plane. In addition, we found for the first time that oculomotor measures (vertical gain, velocity, and the diagnostic index) were correlated significantly to midbrain volume in the PSP group.

**Conclusions:** Assessing eye movements in a HMD provides an easy to apply and highly standardized tool to differentiate PSP of patients from PD and HCs, which will aid in the diagnosis of PSP.

## Introduction

Progressive supranuclear palsy (PSP), first described in 1964 (1), is a 4-repeat (4R) tauopathy that belongs to the group of frontotemporal lobar degeneration disorders (2). The neuropathological diagnosis of PSP is based on the presence of neurofibrillary tangles and threads in subcortical nuclei together with the presence of tufted astrocytes (3–5). In addition, oligodendroglial coiled bodies and diffuse cytoplasmic immunoreactivity in neurons can be observed as well (5–7). The definite diagnosis of PSP requires a neuropathological examination of the brain with the characterization of the tau protein distribution throughout the brain to differentiate PSP from other tauopathies (8). Clinical criteria for the antemortem diagnosis of PSP focus on ocular motor dysfunction, postural instability, akinesia, and cognitive dysfunction (9). The diagnosis of PSP can further be supported by MRI focusing on the regional atrophy in several structures, including the midbrain, thalamus, basal ganglia, insular, and frontal cortex (10–15). From the spectrum of associated symptoms, especially the presence of eye movement abnormalities can be considered as a core feature of the clinical PSP diagnosis (16). Eye movement disorders are a relevant clinical symptom not only for PSP with Richardson's syndrome (PSP-RS), but also in other subtypes of PSP (9) including PSP with progressive gait freezing, PSP with predominant parkinsonism (PSP-P), PSP with predominant frontal presentation, PSP with predominant oculomotor dysfunction, PSP with predominant speech/language disorder, and also PSP with predominant CBS (9). Particularly, vertical saccades are most prominently impaired, showing a decreased velocity and amplitude when compared to related disorders such as Parkinson's disease (PD) (17–20). As the disease progresses, vertical gaze palsy becomes one of the most obvious impairments and, is therefore, often seen as a key cardinal feature for the diagnosis of PSP. However, before vertical supranuclear gaze palsy becomes unequivocally apparent, preliminary signs of eye movement abnormalities (e.g., slowing of vertical saccades) can hardly be detected by conventional bedside testing. Thus, a correct diagnosis of PSP remains challenging in the early stages of the disease, and many patients with PSP are initially thought to suffer from PD (8, 21). For example, at the first clinical visit, the sensitivity to detect PSP is limited, ranging between 14 and 83% with a median of 24% (9). This finding may at least be partially explained by inadequate ocular motor examination.

An adequate eye movement-based diagnosis of PSP demands a high standardization of testing conditions (e.g., room lightning, stimuli, and viewing distance), which is often difficult to achieve across institutions. Moreover, the usage of classical stationary high-resolution video-oculography demands that the patient sits still while the head is fixed. PD and PSP, although typically characterized as hypokinetic movement disorders, are often accompanied by postural instability and facial tremors are not uncommon in PD (22). Thus, the requirement to sit still while the head is fixed often renders stationary video-oculography challenging for the clinical routine testing, in older patients with PD and PSP. A promising solution to enhance standardization and applicability is the high-resolution assessment of eye movements in a head-mounted display (HMD). HMDs offer several benefits since they keep the visual environment constant, allow freedom of movement, are portable, lightweight, and thus, ideally suited for clinical routine (including bedside) testing (23–26). In addition, the video-oculography device of this study allows high-resolution measurements of saccades—a necessary requirement for its reliable assessment.

In this study, we, therefore, investigated if recording eye movements in an HMD using a brief and simple saccade task can differentiate patients with PSP from patients with PD and healthy controls (HCs). These findings were compared to MRI-derived brain volumetry to correlate ocular motor dysfunction and regional brain atrophy.

## Materials and Methods

### Subjects and Study Design

A case–control study was conducted. All tests in subjects assigned to one of the three groups (PSP, PD, and HC) were performed in our hospital, a tertiary referral center.

The PSP group comprised patients diagnosed according to the clinical diagnosis criteria of PSP (9). As controls, patients with PD as well as HCs were included. All the participants with PD met the Movement Disorder Society (MDS) clinical diagnostic criteria (27). HCs were free of neurological, systemic, and psychiatric disease assessed by a neurologist *via* the history of pre-existing conditions and a neurological examination. The HC subjects were not blood-related family members of patients with PD or PSP.

All patients underwent written informed consent about the study, neurological and general medical examination and also assessment of Unified Parkinson's Disease Rating Scale (MDS-UPDRS) (28), Hoehn and Yahr scale (29), and PArkinson Neuropsychometric Dementia Assessment (PANDA) (30) both in patients with Parkinson's syndrome. The selection of patients, information, and medical examinations including the assessment of clinical scores were carried out by a neurologist trained as a specialist in movement disorders. The clinical scores of mobility (Hoehn and Yahr scale; MDS-UPDRS) were assessed in the medical ON condition. Furthermore, the duration of the disease, the levodopa equivalent daily dose (LEDD) (31), and previous performance of a DaTSCAN were identified in the medical history interview and medical records. Before inclusion into the study, participants gave their informed written consent. All procedures conformed to the Declaration of Helsinki and were approved by the local ethics committee of Muenster (file reference: 2018-280-f-S).

### Oculomotor Examination

**Figure 1:**
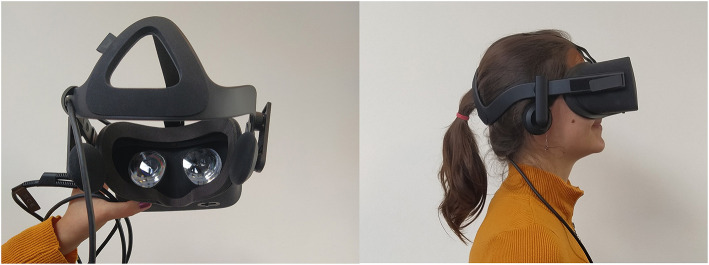
Representation of a head-mounted display (HMD, HTC Vive, New Taipei City, Taiwan) with an integrated binocular eye tracker measuring at 250 Hz (SensoMotoric Instruments). The picture shows a staff member of the working group and thus not a study subject.

We assessed saccadic eye movements in an HMD (HMD, HTC Vive, New Taipei City, Taiwan) with an integrated binocular eye tracker measuring at 250 Hz (SensoMotoric Instruments, Teltow, Germany) (Figure 1). Each test comprised of 192 trials and lasted for about 10–15 min during which subjects had to perform reactive visually guided saccades to vertical or horizontal targets. Each trial of the oculomotor examination started with the appearance of a black central fixation cross (1°) at the center of the screen. The fixation cross turned red as soon as the gaze fall within a rectangular area of 2.2° by 2.2° centered on the fixation cross. After a variable fixation period of 500–1,000 ms, the fixation cross disappeared and a small black open circle (0.5°) appeared as target stimulus at one out of 8 possible locations (left, right, up, or down at 5° or 10°) for 1,000 ms. Trials were separated by an intertrial interval of 1,000 ms. Each of the eight target locations was presented 24 times in a random order and all stimuli were presented against a square white background panel of 50° by 50° presented at a virtual distance of 150 cm. The panel was always shown in front of the participant unaffected by head movements.

Saccade onsets were detected using a built-in detector provided by SensoMotoric Instruments based on velocities. We excluded trials if (a) no saccade was detected, (b) saccades were anticipatory (latency <80 ms), (c) gaze deviated by more than 2.5° from the display center at the time of saccade onset, or (d) saccades amplitude was <1°. With these criteria, 25.8% of trials were discarded from the analysis. There were no significant differences between the three groups in the number of trials discarded.

### Magnetic Resonance Imaging Assessment

The core MRI protocol for all participants comprised three-dimensional (3D) T1-weighted 3D magnetization prepared rapid gradient echo (MPRAGE) sequence with 1 × 1 × 1 mm isotropic voxel size according to the Alzheimer's Disease Neuroimaging Initiative (UCLA, CA, USA; www.loni.ucla.edu/ADNI) recommendations for volumetric analysis, and axial T2-weighted turbo spin-echo sequences (3 mm slice thickness) to detect vascular lesions, acquired on 1.5 or 3T scanners. MR volumetry was performed in analogy to an already published protocols (32).

The MPRAGE sequences were used as input for atlas-based volumetry, a fully automated, observer-independent method for volumetric analysis of MR images using algorithms of SPM12 and masks of diverse brain atlases (33). For this study, we focused on the typical markers of atrophy in PSP, i.e., the midbrain volume and the midsagittal planes of midbrain and midbrain tegmentum (34). All the results were corrected for intracranial volume.

### Statistical Analysis

Statistical analyses were performed using the Statistical Package for the Social Sciences for Windows (Version 26; SPSS, Chicago, Illinois, USA). Oculomotor and MRI data were first examined visually and statistically using the Shapiro-Wilk test for normality across participants. We planned to conduct parametric tests (i.e., ANOVAs, two-tailed *t-*tests, and two-tailed Pearson's correlation) for normally distributed data and non-parametric tests (i.e., Kruskal-Wallis, Mann–Whitney *U*-Test, and Kendall's tau correlation) if the data were non-normally distributed. Data are presented as mean ± SD and a significance criterion of *p* < 0.05 was used for all analyses.

## Results

In total, 54 subjects underwent screening, inclusion in the study, and oculomotor examination. Following exclusion (see later) the PSP group comprised 13 patients; 8 with probable and 5 with possible PSP according to the diagnostic criteria of PSP. As controls, 15 patients with PD as well as 16 HCs were included.

A total of 3 of 15 patients with PD and 11 of 13 patients with PSP underwent SPECT examination with I-123 FP-CIT (DaTSCAN) during the course of their disease (some outside our clinic) and evidence of a reduction in striatal binding quotient compared with a reference region in the occipital cortex. As a reliable differentiation of Parkinson's disease from PSP and other Parkinson's diseases is methodologically not possible using DaTSCAN, a performed examination was not considered a prerequisite for inclusion in the study. The information of the number of performed examinations was presented additionally for the comprehensibility of the study population.

There were no significant differences between the three groups regarding age, gender, and severity of disease measured by the MDS-UPDRS, Hoehn and Yahr scale, and also the LEDD. The duration of the disease and the PANDA scores differed between the PSP and PD groups in that the patients with PSP had a significantly shorter disease duration and higher PANDA scores (see Table 1A). Among the 15 patients with PD, 10 had dyskinesia and 7 had fluctuations. Further details of the characteristics and neurological deficits of patients with PSP are provided in Supplementary Table 2.

**Table 1A:** Clinical characteristics of the subjects in this study.

	**PSP**	**PD**	**HC**	***P*** **-value**
*N*	13	15	16	
Age, mean ± SD	71.9 ± 4.1	73.8 ± 7.9	75.1 ± 10.7	n.s.^a^
Gender, F/M	6/7	7/8	13/3	0.08^b^
Disease duration, mean ± SD	2.4 ± 1.9	9.2 ± 6.0		<0.001^c^
Hoehn and Yahr stage, median	4 (range 2–4)	4 (range 2–4)		n.s.^c^
MDS-UPDRS (complete)	43.6 ± 8.5	37.1 ± 9.2		n.s.^c^
MDS-UPDRS (part III)	27.4 ± 8.6	24.3 ± 6.8		n.s.^c^
PANDA	8.6 ± 8.2	16.3 ± 6.1		0.022^c^
LEDD	552.8 ± 392.0	804.5 ± 327.2		n.s.^c^

### Excluded Subjects

However, 9 subjects (3 PSP, 6 PD) had to be excluded due to insufficient oculomotor data (<40% of valid trials) and 1 additional subject had to be excluded from the HC group due to a former history of stroke. The main reported reasons for discontinuing oculomotor testing were fatigue, problems with vigilance and concentration. The number of discarded trials showed no correlation with age, MDS-UPDRS, PANDA score, and LEDD.

Regarding included and excluded patients with Parkinson's syndrome (PD, PSP), there was no significant difference in age (Mann-Whitney *U*-test 80.5, *Z* = 1.61, *p* = 0.106), gender (Mann-Whitney *U*-test 95.5, *Z* = 1.27, *p* = 0.204), disease duration (Mann-Whitney *U*-test 123.0, *Z* = 0.11, *p* = 0.915), LEDD (Mann-Whitney *U* test 109.5, *Z* = 0.58, *p* = 0.559), PANDA (Mann-Whitney *U*-test 89.0, *Z* = 0.77, *p* = 0.441) as well as the MDS-UPDRS score (Mann-Whitney *U*-test 106.0, *Z* = 0.71, *p* = 0.478).

### Oculomotor Examination

**Figure 2:**
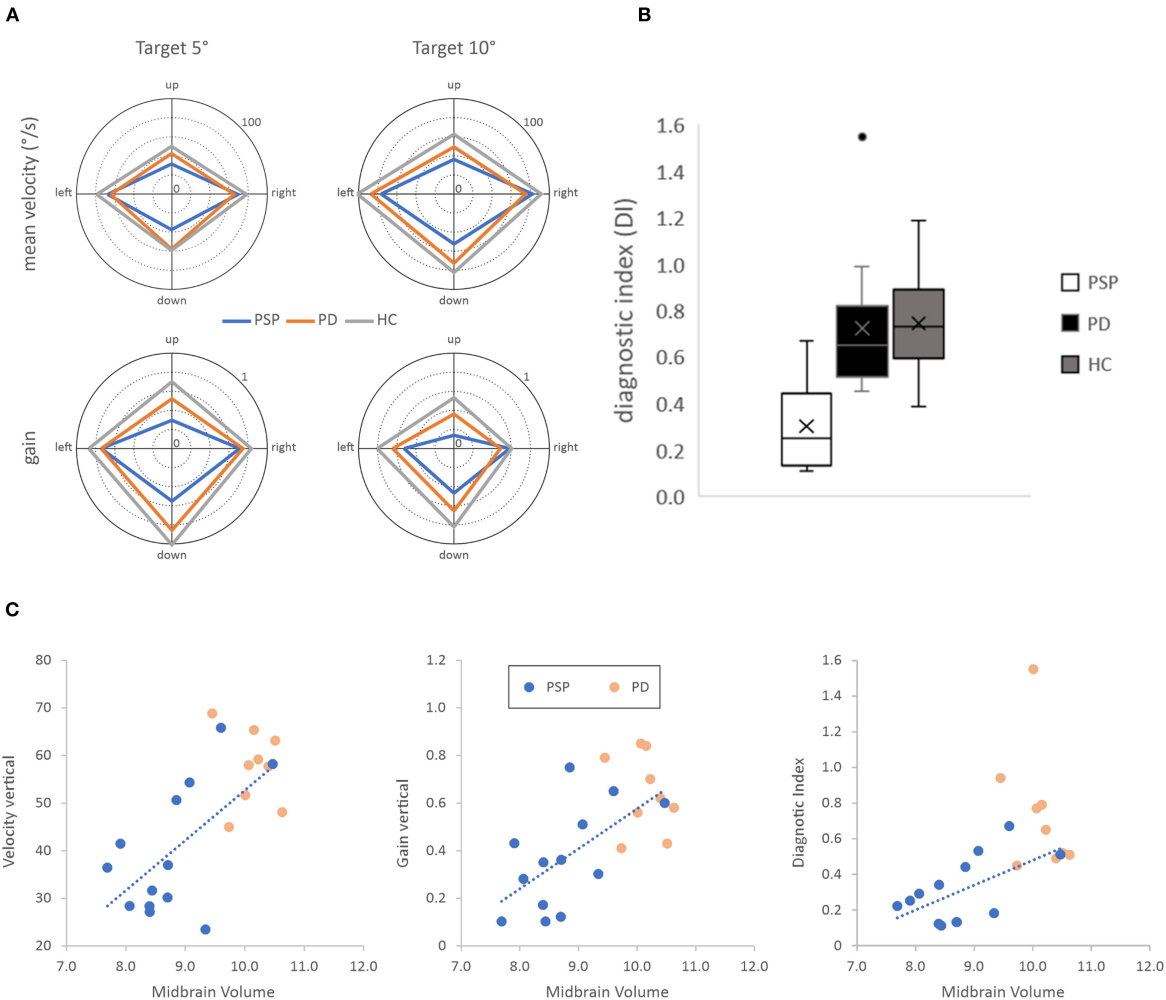
**(A)** Top row, saccadic mean velocity for the three subject groups for targets at 5◦ (left) and 10◦ (right) visual eccentricity. Bottom row, saccadic gain for the three subject groups for targets at 5◦ (left) and 10◦ (right) visual eccentricity. **(B)** Boxplots of the diagnostic index (DI), computed as the product of the ratios of vertical to horizontal gain and velocity, for the three subject groups. **(C)** Scatterplots show significant correlations between oculomotor parameters and midbrain volume in the progressive supranuclear palsy (blue dots) but not the Parkinson's disease (orange dots) group.

For each trial of the oculomotor examination, we analyzed the mean velocity and gain (the ratio of saccadic amplitude and target distance) of the first saccade after target presentation as a function of *group* (PSP vs. PD vs. HC), *eccentricity* (5 vs. 10°), and *direction* (left vs. right vs. up vs. down), all of which are depicted in Figure 2A. For statistical analysis, we further collapsed the data across eccentricity and the horizontal (i.e., left and right) as well as the vertical (i.e., up and down) direction and ran a 2 (direction*:* horizontal vs. vertical) x 3 (group*:* PSP vs. PD vs. HC) mixed ANOVA on the velocity and gain data.

Analysis of the mean saccadic velocity revealed a significant main effect of direction, *F*_(1, 41)_ = 213.985, *p* < 0.001, $\eta_{\text{p}}^{2}$ = 0.839, confirming that mean velocity was lower for vertical (53.7 ± 15.5°/s) than horizontal (78.5 ± 17.1°/s) saccades. Moreover, there was also a significant main effect of group, *F*_(2, 41)_ = 7.741, *p* < 0.01, $\eta_{\text{p}}^{2}$ = 0.274, showing that mean velocity was lower for patients with PSP (56.6 ± 14.6°/s) than for patients with PD (64.2 ± 12.3°/s) and HCs (75.5 ± 12.4°/s). Importantly, both main effects were qualified by a significant interaction of group and direction *F*_(2, 41)_ = 7.827, *p* < 0.01, $\eta_{\text{p}}^{2}$ = 0.276. *Post-hoc t*-test showed that the PSP group and the PD group differed in the mean velocity of vertical [PSP < PD, *t*_(26)_ = −3.55, *p* < 0.01; PD = HC, *t*_(29)_ = −1.93, *p* > 0.05] but not horizontal saccades [PSP = PD, *t*_(26)_ = −0.14, *p* > 0.05; PD < HC, *t*_(29)_ = −2.68, *p* < 0.05].

The analysis of saccadic gain revealed a similar pattern. There was also a significant main effect of direction, *F*_(1, 41)_ = 10.172, *p* < 0.01, $\eta_{\text{p}}^{2}$ = 0.199, confirming that saccadic gain was lower for vertical (0.59 ± 0.25) than horizontal (0.70 ± 0.17) saccades. Once again, there was a significant main effect of group, *F*_(2, 41)_ = 15.743, *p* < 0.001, $\eta_{\text{p}}^{2}$ = 0.434, showing that gain was lower for patients with PSP (0.50 ± 0.19) than for patients with PD (0.63 ± 0.17) and HCs (0.77 ± 0.13). Importantly, both main effects were qualified by a significant interaction of group and direction *F*_(2, 41)_ = 7.752, *p* < 0.01, $\eta_{\text{p}}^{2}$ = 0.274. *Post-hoc t*-test showed that the PSP group and the PD group differed in the mean velocity of vertical [PSP < PD: *t*_(26)_ = −2.97, *p* < 0.01; PD < HC: *t*_(29)_ = −2.50, *p* < 0.05] but not horizontal saccades [PSP = PD: *t*_(26)_ = −0.22, *p* > 0.05; PD < HC: *t*_(29)_ = −2.24, *p* < 0.05].

Based on these findings, we developed a novel *diagnostic index* multiplying the ratios of vertical-to-horizontal velocity and gain to differentiate patients with PSP from patients with PD and HCs. This index is calculated as follows:


$$\begin{array}{l} diagnostic\;index=\frac{Velocity_{vertical}}{Velocity_{horizontal}}\;*\;\frac{Gain_{vertical}}{Gain_{horizontal}} \end{array}$$

The diagnostic index differed significantly between groups, *F*_(2, 41)_ = 14.765, *p* < 0.001, $\eta_{\text{p}}^{2}$ = 0.419, PSP < PD: *t*_(26)_ = −4.73, *p* < 0.001, PSP < HC: *t*_(27)_ = −5.64, *p* < 0.001, PD = HC: *t*_(29)_ = −0.22, *p* > 0.05 (see Figure 2B). To assess the diagnostic ability of the diagnostic index, we further performed signal-detection analysis by computing the receiver-operating characteristic (ROC) which can be quantified by its area under the curve (AUC), the cut-off point for maximal specificity and sensitivity, and the corresponding values of specificity and sensitivity. The ROC comparing the diagnostic index of the PSP and the combined group of PD-HC showed an AUC of 0.95. Specificity was 28/31 and sensitivity was 10/13 for the cut-off value of 0.47. ROC analysis comparing the diagnostic index of the PSP to the PD group revealed an AUC of 0.96 with a specificity of 14/15 and sensitivity was 10/13 for the cut-off value of 0.47.

### Magnetic Resonance Imaging Assessment

Data of MRI were obtained from all patients of the PSP group and 10 out of 15 patients of the PD group. The clinical characteristics of the patients with MRI data are shown in Table 1B. Moreover, Table 1C shows the data of different brain structures in these patients. Analysis revealed that patients with PSP displayed significantly lower midbrain volume. Moreover, the midbrain plane, as well as the midbrain tegmentum plane, were also significantly decreased in patients with PSP as compared with patients with PD.

**Table 1B:** Clinical characteristics of the subsample with MRI assessment.

	**PSP**	**PD**	***P*** **-value**
*N*	13	10	
Age, mean ± SD	71.9 ± 4.1	72.7 ± 8.6	n.s.^a^
Gender, F/M	6/7	5/5	n.s.^b^
Disease duration, mean ± SD	2.4 ± 1.9	9.0 ± 6.0	<0.001^c^
Hoehn and Yahr stage, median	4 (range 2–4)	4 (range 2–4)	n.s.^c^
MDS-UPDRS (complete)	43.6 ± 8.5	36.1 ± 9.8	n.s.^c^
MDS-UPDRS (part III)	27.4 ± 8.6	23.9 ± 7.3	n.s.^c^
PANDA	8.6 ± 8.2	16.9 ± 6.1	0.030^c^
LEDD	552.8 ± 392.0	795.3 ± 379.0	n.s.^c^

**Table 1C:** Volumes (ml) and areas (mm^2^) of different brain structures.

	**PSP**	**PD**	***P*** **-value**
Midbrain volume	8.74 ± 0.76	10.19 ± 0.38	<0.0001^a^
Midbrain plane	247.58 ± 50.72	286.13 ± 40.81	<0.01^c^
Midbrain tegmentum plane	139.53 ± 25.15	168.40 ± 16.45	<0.01^a^

a *Student t-test, ^b^Chi-squared and*

c *Mann–Whitney U-test used as appropriate.*

*PSP, progressive supranuclear palsy; PD, Parkinson's disease; HC, healthy controls; F, female; M, male; LEDD, levodopa equivalent daily dose; n.s., not significant*.

### Correlational Analysis

Based on the significant differences between patients with PSP and PD observed for the oculomotor (i.e., vertical gain, vertical velocity, and diagnostic index) and MRI assessment (i.e., the midbrain volume, midbrain plane, and midbrain tegmentum plane) we analyzed the relationship between oculomotor performance and MRI data separately for the PSP and also the PD group in a correlational analysis (see Supplementary Table 1B; Figure 2C). This analysis revealed significant and high-positive correlations of vertical gain (*r* = 0.607), vertical velocity (*r* = 0.596), and the diagnostic index (*r* = 0.592) with the midbrain volume in the PSP but not the PD group. Moreover, Fischer r-to-z transformation revealed that correlation coefficients significantly differed between both groups for the diagnostic index (*Z* = 2.25, *p* = 0.024) but not for vertical velocity (*Z* = 1.73, *p* = 0.084) and vertical gain (*Z* = 1.59, *p* = 0.112).

## Discussion

The investigation of movements of the human eye has evolved steadily over the past 40 years and has become an important tool in the study of cognitive dysfunction and pathophysiology in neurological disorders, including Parkinson's disease. In addition to measuring saccadic eye movements (35–38), examination of eye blinking abnormalities has provided further insights into pathophysiological correlates of PSP (39, 40). For example, patients with PSP showed a prolonged blink duration during voluntary blinking that was inversely correlated with volume loss in the left putamen and caudate (41). Eye-tracking has thus established itself as a method to objectively measure the consequences of neurological damage and is now an important diagnostic tool to study disease progression and prognosis. Whereas, eye movements used to be recorded using stationary devices that left little freedom of movement patients, modern eye trackers have become smaller and more mobile. As a consequence, wearable devices for gait or oculomotor analysis have started to further assist and facilitate diagnostic procedures in PSP and PD (42–44).

In this study, we tested for the first time a novel technology of eye-tracking in HMDs to assess gaze behavior in patients with PSP, PD, and HCs. Besides easy handling and portability, HMDs offer the additional benefit of an inherently standardized visual environment making the visual stimulation exclusively dependent on the programmed environment (23, 24). In addition, this novel technology is becoming increasingly affordable, as there are currently several companies offering eye trackers for HMDs (45). Thus, the first clear advantage of HMDs over other methods is the capability of a highly standardized oculomotor assessment across clinical institutions. This is an important prerequisite to decisively improve the reliability of oculomotor diagnostics. The second clear advantage is that the assessment of eye movements in HMDs does not require testing in restrained conditions or body postures making it thus ideally suited for bedside testing. Using this novel technology, patients with PSP displayed unique oculomotor abnormalities concerning the velocity and the gain of vertical saccades. Our results of the oculomotor examination in HMD are thus highly consistent with earlier findings observed in standard laboratory setups (17–20), showing for the first time that eye-tracking in HMDs is comparable to classical stationary video-oculography in PSP.

We further crucially extended this finding by developing a new diagnostic index combining the ratios of vertical to horizontal gain and velocity in one single measurement. In our sample, the diagnostic index distinguished PSP from PD with high sensitivity and specificity. That is, patients with a diagnostic index below 0.47 were highly likely to suffer from PSP whereas almost all subjects with an index above 0.47 were either patients with PD or HCs. We see the decisive advantage of this new index especially in the diagnosis of PSP during early stages of the disease before impairments of vertical saccades are followed by impairments of horizontal saccades (46). Moreover, during early stages, there is only slight impairment of vertical saccades and considerable difficulty in the diagnostic assignment. The method may, thus, contribute an objective assessment of vertical saccade motion disturbance. This could provide additional diagnostic value, especially for the less experienced examiner. In this regard, prospective studies evaluating the diagnostic index of as yet unclassified patients may be of interest and desire in the future to further evaluate whether subclinical oculomotor disorders are captured before the clinical diagnosis is made. Our patients were those who already had abnormalities of oculomotor function on neurologic examination by an experienced neurologist focused on Parkinson's syndromes. The question of whether subclinical abnormalities can already be detected cannot be proven within the framework of this study design, but can only be clarified prospectively. In this context, supplemental validation of the threshold derived from the ROC analysis in this study will also be required in an independent population of patients.

The results of the MRI assessment confirmed regional atrophy in PSP in the midbrain volume and the midbrain as well as the midbrain tegmentum plane (10–15). Several MRI studies on PSP did not observe a correlation between MRI measurements and clinical scores (14, 47, 48). Importantly, we found vertical gain and velocity as well as the new diagnostic index to correlate to the midbrain volume in the PSP group but not in the PD group. Moreover, correlations significantly differed between both groups for the diagnostic index. To our knowledge, this is the first observation of a significant correlation between the degree of oculomotor dysfunction and midbrain atrophy in PSP.

## Study Limitations

This study has some potential limitations. First, the PSP and the PD group differed in disease duration. Despite the shorter disease duration patients with PSP showed unique oculomotor dysfunctions and patients with PD with a longer disease duration resembled the group of HCs. Second, a relatively large number of patients (9/54 = 17%) were not able to complete the oculomotor examination either due to reported fatigue or problems with vigilance and concentration. These patients had to be excluded because the number of valid trials was too low. This problem might be reduced by either shortening the oculomotor examination or by retesting. However, we verified that no systematic bias occurred by excluding patients demonstrating the absence of significant differences between included and excluded patients with regard to age, gender, disease duration, LEDD, mental assessment using PANDA as well as motor status using MDS-UPDRS score.

It must be mentioned that a large number of the patients in our cohort had possible or probable PSP of the Steele–Richardson type. In many of these patients, disturbances of vertical eye movements could already be detected in the clinical examination, so our method validated the clinical finding. We believe, however, that our method has the potential to identify early stages of the disease when oculomotor symptoms are not yet visible. Further studies should therefore focus on the evaluation of abnormalities in the early phase of still inconspicuous clinical eye movements to improve subtype diagnosis. For patients with PSP, the clinical assessment was done using MDS-UPDRS rather than the Progressive Supranuclear Palsy Rating Scale (PSP-RS) or the newer Progressive Supranuclear Palsy Clinical Deficits Scale (PSP-CDS). In further studies using this method, the additional collection of these scores would be useful for further assessment of a possible association between the midbrain atrophy and clinical severity.

## Conclusion

The assessment of eye movements in HMDs provides a new, convenient, and highly standardized tool to differentiate patients with PSP from PD and HCs. Due to the high level of standardization, this novel technology has the potential to significantly improve the reliability of oculomotor diagnostics in the clinical setting. Our easy to compute diagnostic index differentiates PSP from PD and might especially enhance diagnostics during the early stages of PSP. Finally, we have provided evidence for the first time that the degree of vertical ocular dysfunction correlates with the severity of midbrain atrophy. However, at this stage of development, patient exclusion due to discontinuation of oculomotor examination is still evident as well as the diagnostic discriminatory power in the early phase of the disease, which remains to be prospectively investigated. Further prospective and longitudinal investigations on a broader number of samples and timepoints are therefore mandatory to comprehensively assess the utility of eye-tracking in PSP diagnostics.

## Data Availability Statement

The raw data supporting the conclusions of this article will be made available by the authors, upon reasonable request.

## Ethics Statement

The studies involving human participants were reviewed and approved by local Ethics Committee of Muenster (file reference: 2018-280-f-S). The patients/participants provided their written informed consent to participate in this study.

## Author Contributions

AH, WS, W-RS, and AR designed the study and drafted the manuscript. AH, AA, and AR performed data acquisition. AH and AR performed statistical analysis. H-JH and RK performed MRI analysis. FZ, H-JH, and RK revised the manuscript. All authors contributed to the article and approved the submitted version.

## Funding

This study has been funded by a grant to Werner Schneider from the Cluster of Excellence Cognitive Interaction Technology CITEC (EXC 277, German Research Foundation) at Bielefeld University.

## Conflict of Interest

The authors declare that the research was conducted in the absence of any commercial or financial relationships that could be construed as a potential conflict of interest.

## Publisher's Note

All claims expressed in this article are solely those of the authors and do not necessarily represent those of their affiliated organizations, or those of the publisher, the editors and the reviewers. Any product that may be evaluated in this article, or claim that may be made by its manufacturer, is not guaranteed or endorsed by the publisher.
