# Supplementary material for: Differentiating Progressive Supranuclear Palsy and Parkinson's Disease With Head-Mounted Displays
Source: Front Neurol. 2021 Dec 23;12:791366. doi: 10.3389/fneur.2021.791366 (PMC8733559; doi:10.3389/fneur.2021.791366)
Supplement: Supplementary file 1 [file Table_1.docx]

**Supplementary Table 1.** Correlations between oculomotor parameters and MRI-parameters for the PSP and PD group

| Occulomotor parameters | MRT-Assessment | | | | | |
| --- | --- | --- | --- | --- | --- | --- |
|  | PSP (N=13) | | | PD (N=10) | | |
|  | MBV^a^ | MBP^b^ | MBTP^a^ | MBV^a^ | MBP^b^ | MBTP^a^ |
| Vel_vertical | **0.596 (.031)** | 0.154 (.464) | 0.188 (.539) | -0.132 (.715) | 0.111 (.655) | 0.017 (.962) |
| Gain_vertical | **0.607 (.028)** | 0.205 (.329) | 0.098 (.750) | -0.048 (.895) | 0.244 (.325) | 0.179 (.620) |
| DI | **0.592 (.033)** | 0.231 (.272) | 0.139 (.649) | -0.368 (.295) | 0.156 (.531) | -0.187 (.605) |

^a^Pearson’s correlation and ^b^Kendall Tau correlation used as appropriate. Results are expressed as r values (p values). PSP, progressive supranuclear palsy; PD, Parkinson’s disease; MBV, midbrain volume; MBP, midbrain plane; MBTP, midbrain tegmentum plane; Vel, velocity; DI, diagnostic index.
