# Supplementary material for: Differentiating Progressive Supranuclear Palsy and Parkinson's Disease With Head-Mounted Displays
Source: Front Neurol. 2021 Dec 23;12:791366. doi: 10.3389/fneur.2021.791366 (PMC8733559; doi:10.3389/fneur.2021.791366)
Supplement: Supplementary file 2 [file Table_2.docx]

**Supplementary Table 2.** Characteristics and neurological deficits of PSP patients.

The operationalised definitions of key clinical features, supporting clinical clues and supporting imaging findings have been listed in brackets according to the definitions of Höglinger et al.(9)

The listed LEDD indicates the levodopa equivalent dose on the day of the eye-tracking examination.

Levodopa resistance was defined as improvement of the MDS-UPDRS motor scale by ≤30%; to fulfill this criterion patients should be assessed having been given at least 1,000 mg (if tolerated) at least 1 month OR once patients have received this treatment they could be formally assessed following a challenge dose of at least 200 mg. (9).

| **Age, gender** | **DD** | **LEDD** | **Neurological syndrome** | **Diagnosis** |
| --- | --- | --- | --- | --- |
| 72, male | 1 year | 400 mg | vertical supranuclear palsy (O1), repeated unprovoked falls (P1), left-lateralized,  akinetic-rigid Parkinsonism (A1), MCI. Levodopa resistance (CC1), midbrain atrophy and reduced Volume of the upper cerebellar peduncles (IF1), pathological DaTSCAN^TM^ | probable PSP-P |
| 70, female | 2 years | 1000 mg | slow velocity of vertical saccades (O2), more than 2 steps backward on pulltest (P3), Left-lateralized Parkinsonism (A1), cognitive dysfunction (C2), Levodopa resistance up to 1000 mg LEDD (CC1), predominant midbrain atrophy (IF1), pathological DaTSCAN^TM^. | possible PSP-RS |
| 73, female | 1 year | 100 mg | slow velocity of vertical saccades (O2), no falls, no postural instability. Right-lateralized Parkinsonism (A1), cognitive fronto-temporal dysfunction (C2) Levodopa resistance (CC1), predominant midbrain atrophy (IF1), pathological DaTSCAN^TM^. | possible PSP-RS |
| 78, male | 2 years | 200 mg | vertical supranuclear palsy (O1), no falls, no postural instability. Left-lateralized akinetic-rigid Parkinsonism (A1), relevant word finding disorders with avoidance of empty phrases (C1). Mild cognitive impairment (MCI). Levodopa resistance (CC1). Hypokinetic, spastic dysarthria (CC2), pathological DaTSCAN^TM^. | possible PSP-SL |
| 71, male | 6 years | 915 mg | vertical supranuclear palsy (O1), repeated unprovoked falls within 3 years (P1),  Parkinsonism, akinetic-rigid, predominantly axial, and levodopa resistant (A2, CC1). Cognitive fronto-temporal dysfunction (C2). Hypokinetic, spastic dysarthria (CC2), Dysphagia (CC3). Predominant midbrain atrophy (IF1), pathological DaTSCAN^TM^. | probable PSP-RS |
| 75, male | 2 years | 400 mg | vertical supranuclear palsy (O1), repeated unprovoked falls within 3 years (P1), Left-lateralized Parkinsonism (A1), MCI. Levodopa resistance (CC1). Hypokinetic, spastic dysarthria (CC2), Dysphagia (CC3). Predominant midbrain atrophy (IF1), no DaTSCAN^TM^ performed. | probable PSP-RS |
| 63, male | 3 years | 1000 mg | vertical supranuclear palsy (O1), no falls, no postural instability. Bilateral akinetic-rigid Parkinsonism (A1), cognitive fronto-temporal dysfunction (C2). Reduced word fluency and naming capability. Levodopa resistance (CC1). Hypokinetic, spastic dysarthria (CC2), pathological DaTSCAN^TM^. | possible PSP-SL |
| 67, male | 1 year | 150 mg | slow velocity of vertical saccades (O2), repeated unprovoked falls within 3 years (P1), Right-lateralized Parkinsonism (A1). Impairment of language skills, executive functions, and visuo-constructive abilities (C2). Levodopa resistance (CC1). Hypokinetic, spastic dysarthria (CC2), pathological DaTSCAN^TM^. | possible PSP-SL |
| 77, male | 7 years | 1121 mg | vertical supranuclear palsy (O1), repeated unprovoked falls within 3 years (P1), Bilateral akinetic-rigid Parkinsonism (A1). Slow flow of speech with word finding Disturbances and iterations of words, syllables and phonemes (C2). Levodopa resistance (CC1). Hypokinetic, spastic dysarthria (CC2), Dysphagia (CC3). mesencephalic atrophy with distinct hummingbird sign, temporomesial atrophy (IF1), no DaTSCAN^TM^ performed. | probable PSP-RS |
| 74, female | 2 years | 1000 mg | vertical supranuclear palsy (O1), repeated unprovoked falls within 3 years (P1), Bilateral akinetic-rigid Parkinsonism (A1). Cognitive dysfunction. Levodopa resistance (CC1). Mild dysarthria, pathological DaTSCAN^TM^. | probable PSP-RS |
| 73, female | 2 years | 400 mg | slow velocity of vertical saccades (O2), tendency to fall on pulltest (P2). Bilateral akinetic-rigid Parkinsonism (A1). Mild cognitive impairment (MCI). Levodopa resistance (CC1). Mild rigid-hypokinetic dysarthrophonia with impaired prosody and phonation. Mild midbrain atrophy and atrophy of the frontal lobe (IF1), pathological DaTSCAN^TM^. | probable PSP-RS |
| 73, female | 1 year | 400 mg | slow velocity of vertical saccades (O2), repeated unprovoked falls within 3 years (P1). Right-lateralized Parkinsonism (A1). Levodopa resistance (CC1), pathological DaTSCAN^TM^. | probable PSP-RS |
| 69, female | 1 year | 100 mg | slow velocity of vertical saccades (O2), tendency to fall on pulltest (P2). Bilateral akinetic-rigid Parkinsonism (A1). Levodopa resistance (CC1). Mild rigid-hypokinetic dysarthrophonia. Midbrain atrophy (IF1), pathological DaTSCANTM. | probable PSP-RS |

DD, disease duration; LEDD, levodopa equivalent daily dose; MCI, mild cognitive impairment

PSP-P, PSP with predominant parkinsonism; PSP-RS, PSP with Richardson’s syndrome; PSP-SL, PSP with predominant speech/language disorder

Comment regarding DaTSCAN^TM^: As a reliable differentiation of Parkinson's disease from PSP and other Parkinson's diseases is methodologically not possible using DaTSCAN^TM^, a performed examination was not considered a prerequisite for inclusion in the study. The information of the number of performed examinations was presented additionally for the comprehensibility of the study population.
